# The Successful Resolution of Bilateral Recurrent Shoulder Dislocation With the Bilateral Shoulder Open Latarjet Procedure: A Case Report

**DOI:** 10.7759/cureus.50569

**Published:** 2023-12-15

**Authors:** Kumarendran Kanesen, Raymond Dieu Kiat Yeak, Johan Abdul Kahar, Mohd Nizlan Mohd Nasir

**Affiliations:** 1 Orthopaedic Surgery, Hospital Sultan Abdul Aziz Shah, University Putra Malaysia, Serdang, MYS

**Keywords:** anterior glenoid rim, recurrent shoulder instability, open surgery, latarjet procedure, bilateral recurrent shoulder dislocation

## Abstract

Recurrent shoulder dislocation is a common orthopedic condition, but bilateral involvement is rare and presents unique challenges in management. The Latarjet procedure is an effective surgical technique that addresses instability by creating a bony block on the anterior glenoid rim. This case highlights the successful management of bilateral recurrent shoulder dislocation using the bilateral shoulder open Latarjet procedure and emphasizes the importance of early intervention in such cases.

## Introduction

Recurrent shoulder dislocation is a debilitating condition that significantly affects the quality of life of affected individuals. Bilateral involvement is rare but can lead to severe functional limitations [1]. While conservative management is typically attempted initially, surgical intervention may be required in cases of recurrent instability. The Latarjet procedure, introduced by Michel Latarjet in 1954, involves the transplantation of the coracoid process to the scapular neck. This surgical technique has not only demonstrated excellent long-term clinical outcomes but also remarkable return-to-sport rates [1]. It has emerged as a reliable method for managing recurrent shoulder dislocations [1]. This case report describes the successful treatment of bilateral recurrent shoulder dislocation using the bilateral shoulder open Latarjet procedure.

## Case presentation

A 24-year-old male patient, a boxing instructor by profession, presented to our orthopedic clinic with a complaint of recurrent shoulder dislocation in both shoulders. The patient reported that the initial injury occurred during a football match five years ago when he fell onto his outstretched arms after being tackled by an opponent team member, resulting in a left anterior shoulder dislocation. A few months later, the patient experienced a right anterior shoulder dislocation when he fell down a staircase while using his outstretched arms to break the fall. Following this incident, he experienced recurrent episodes of shoulder dislocation in both shoulders during various physical activities, including boxing training sessions. The patient reported severe pain, loss of function, and instability in both shoulders. Each dislocation episode required manual reduction at the emergency department, and in some instances, the patient was able to self-reduce the dislocated shoulder. Despite attempts at conservative management, including immobilization and physiotherapy, the patient continued to experience recurrent dislocations, significantly impacting his professional and personal life.

**Figure 1:**
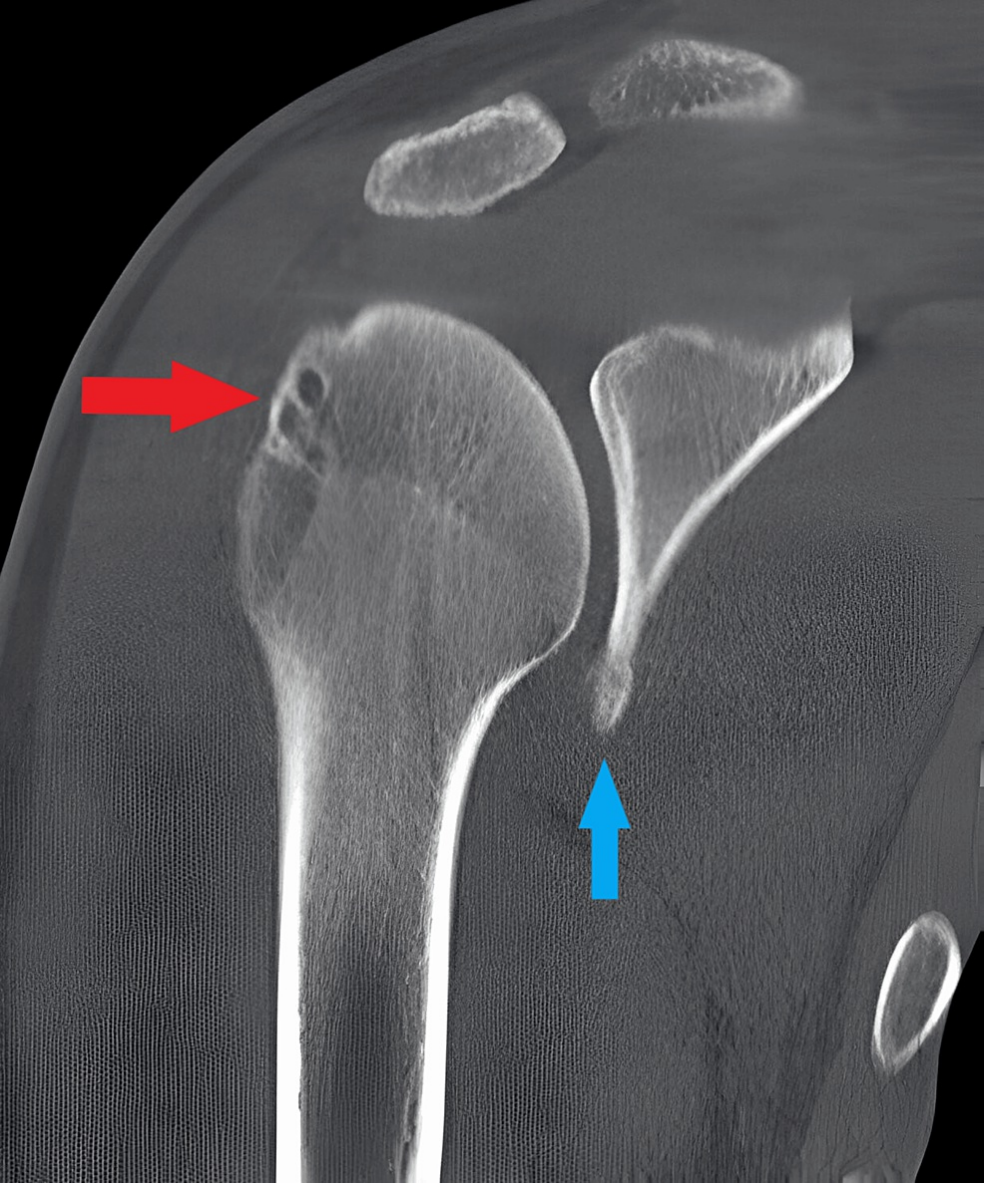
CT scan coronal view of right shoulder showing Bankart lesion (blue arrow) and Hill-Sach lesion (red arrow).

**Figure 2:**
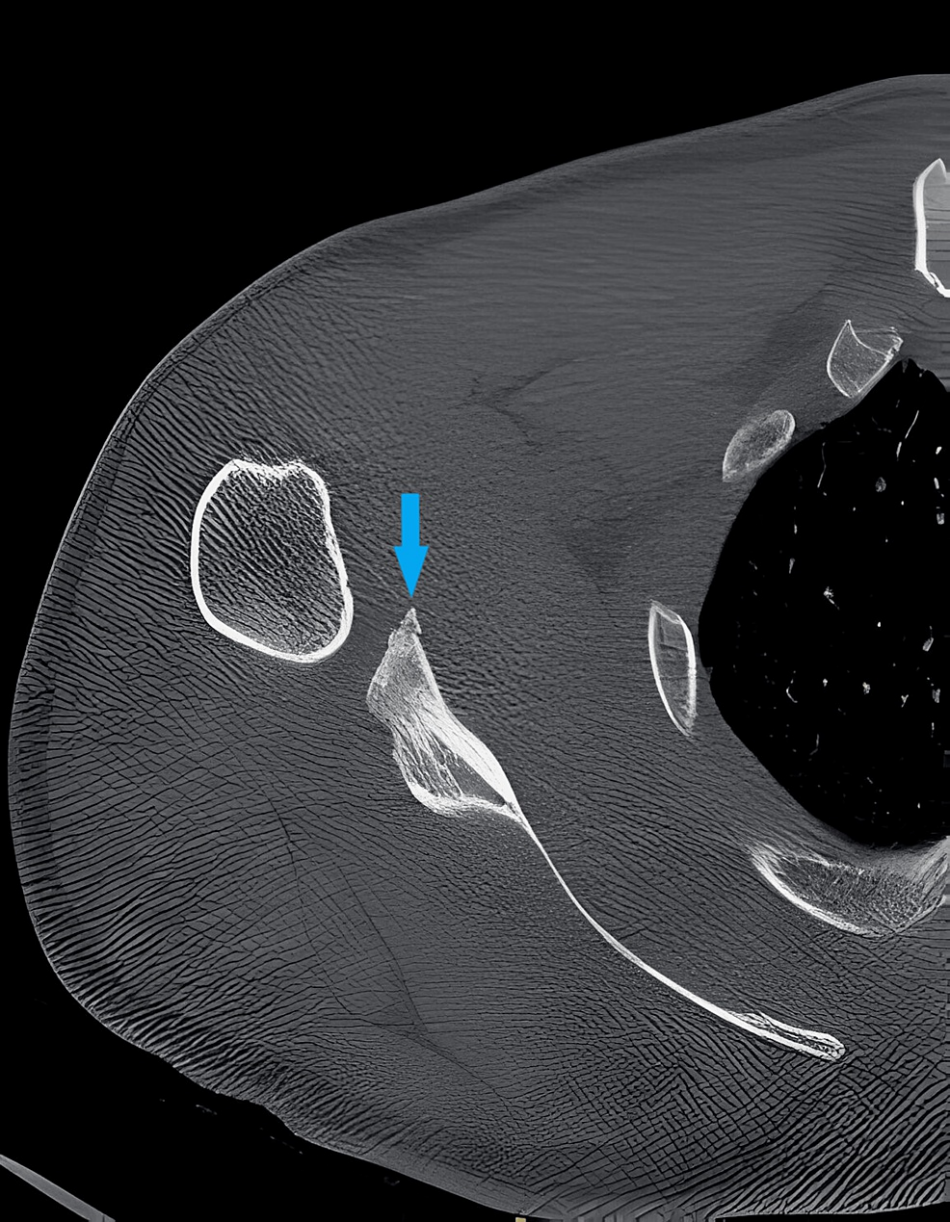
CT scan axial view of right shoulder showing bony Bankart lesion (blue arrow).

**Figure 3:**
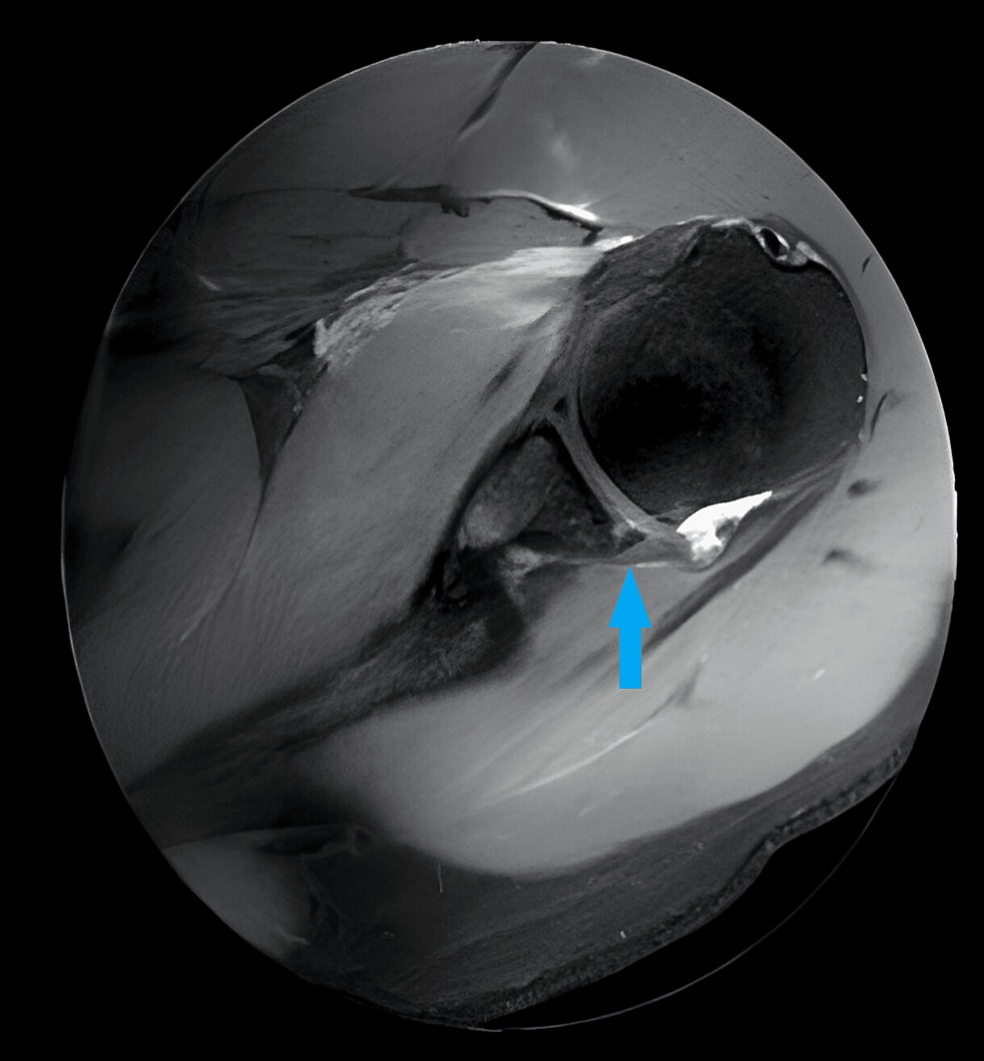
MRI scan T2-weighted axial view of right shoulder showing bony Bankart lesion (blue arrow).

On physical examination, bilateral shoulder laxity was observed, with positive apprehension, relocation tests, and anterior drawer tests indicating anterior instability. The range of motion was limited due to pain and apprehension. Neurovascular examination revealed no abnormalities. Radiographic evaluation, including anteroposterior and scapular Y views, as well as CT scans of both shoulders, revealed bilateral glenoid bone loss of less than 20% along with Hill-Sachs lesions (Figures 1, 2). MRI further confirmed the presence of anterior labral tears, Bankart lesions, Hill-Sachs lesions, and associated soft tissue injuries in both shoulders (Figure 3). Based on the patient's clinical presentation, history of recurrent dislocations, and radiographic findings, a diagnosis of bilateral recurrent shoulder dislocation with concurrent glenoid bone loss and Hill-Sachs lesions was established.

Given the bilateral nature of the shoulder instability and the presence of glenoid bone loss over bilateral shoulder, surgical intervention was considered the most appropriate treatment option for this patient. After a detailed discussion of the surgical options, risks, and potential benefits, the patient provided informed consent for a bilateral shoulder open Latarjet procedure. However, due to the left shoulder instability being more prominent and causing greater functional impairment, the patient decided to undergo the procedure on the left shoulder first. Subsequently, the right shoulder was addressed in a separate surgical setting one month later.

**Figure 4:**
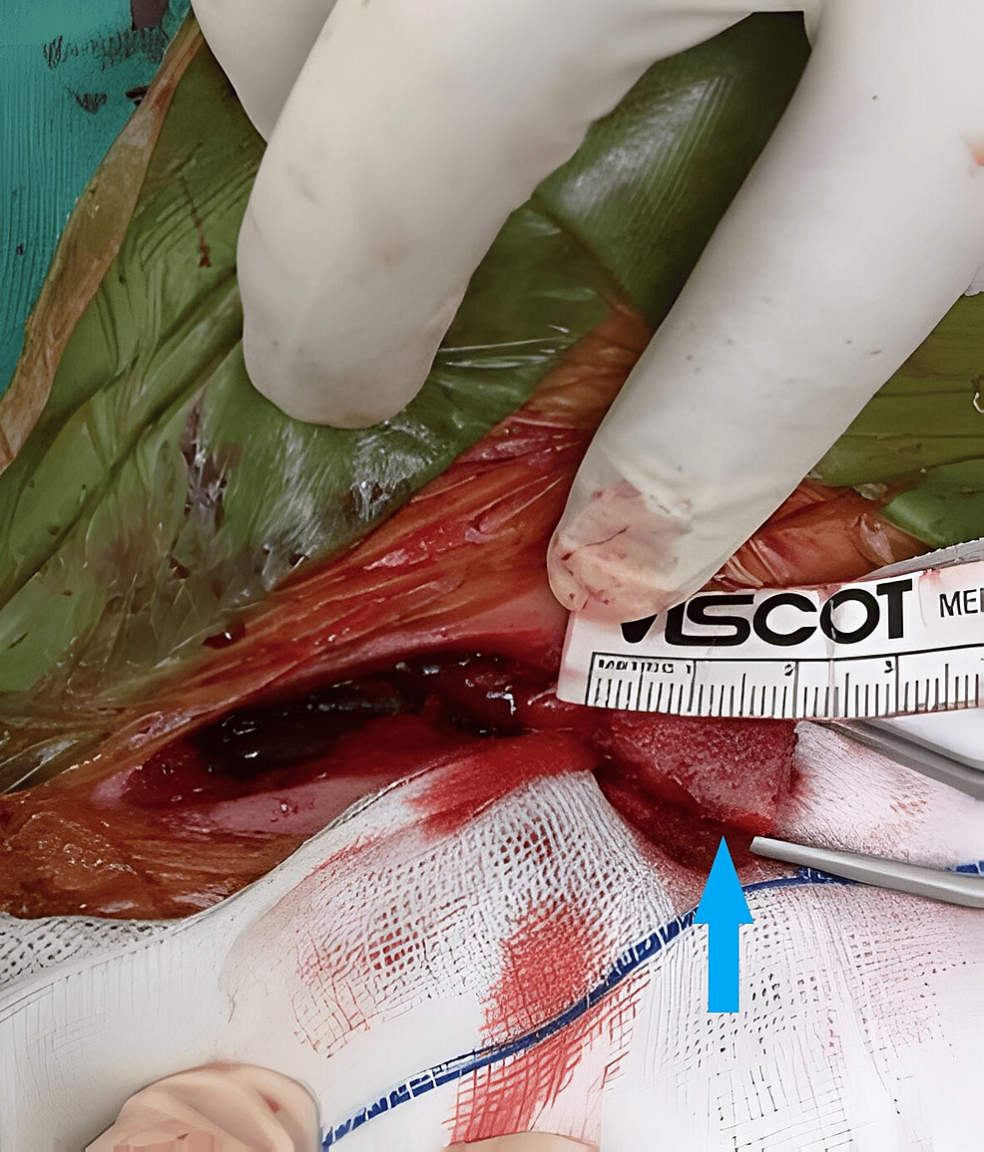
Intraoperative image showing harvested coracoid process measuring 2cm in length (blue arrow).

**Figure 5:**
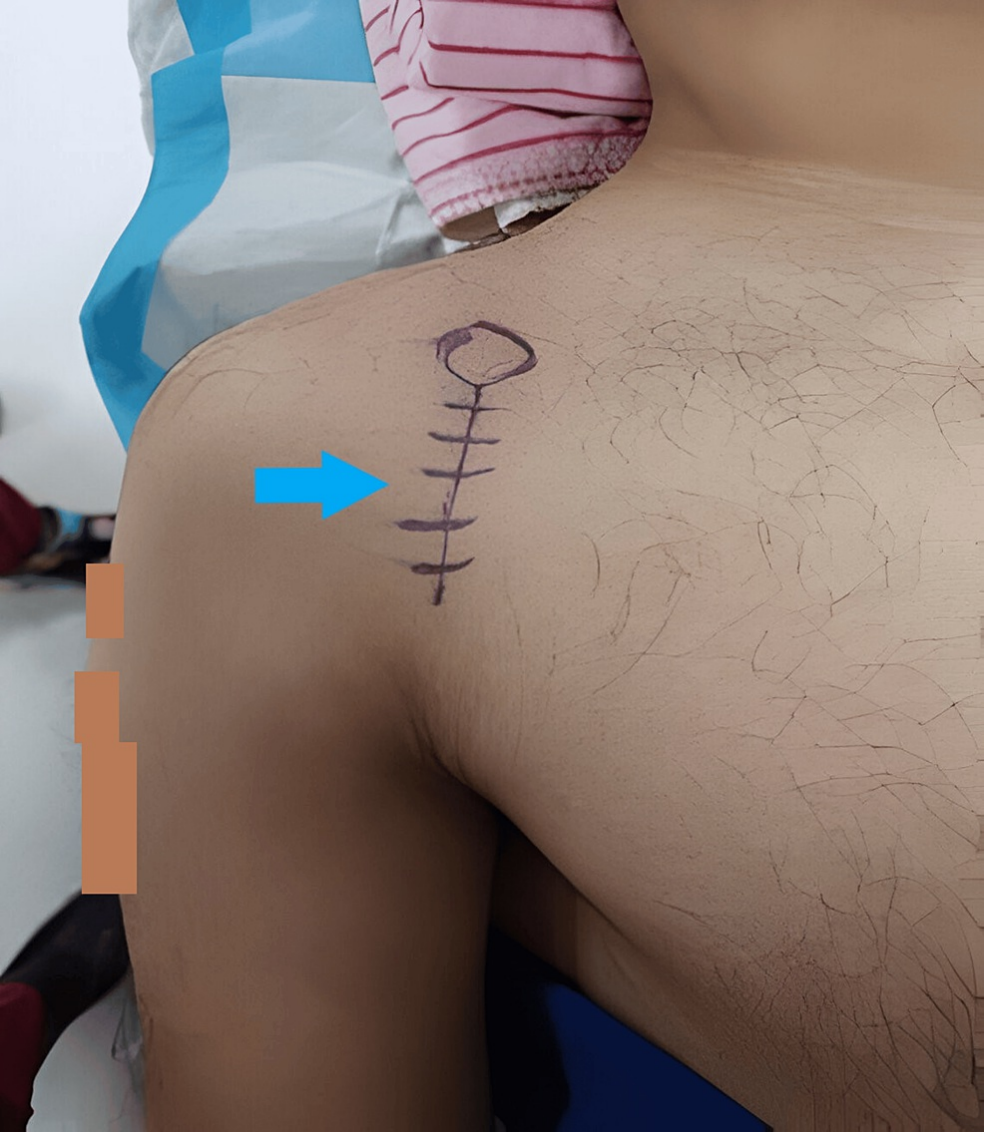
Intraoperative surgical site marking using deltopectoral approach (blue arrow).

**Figure 6:**
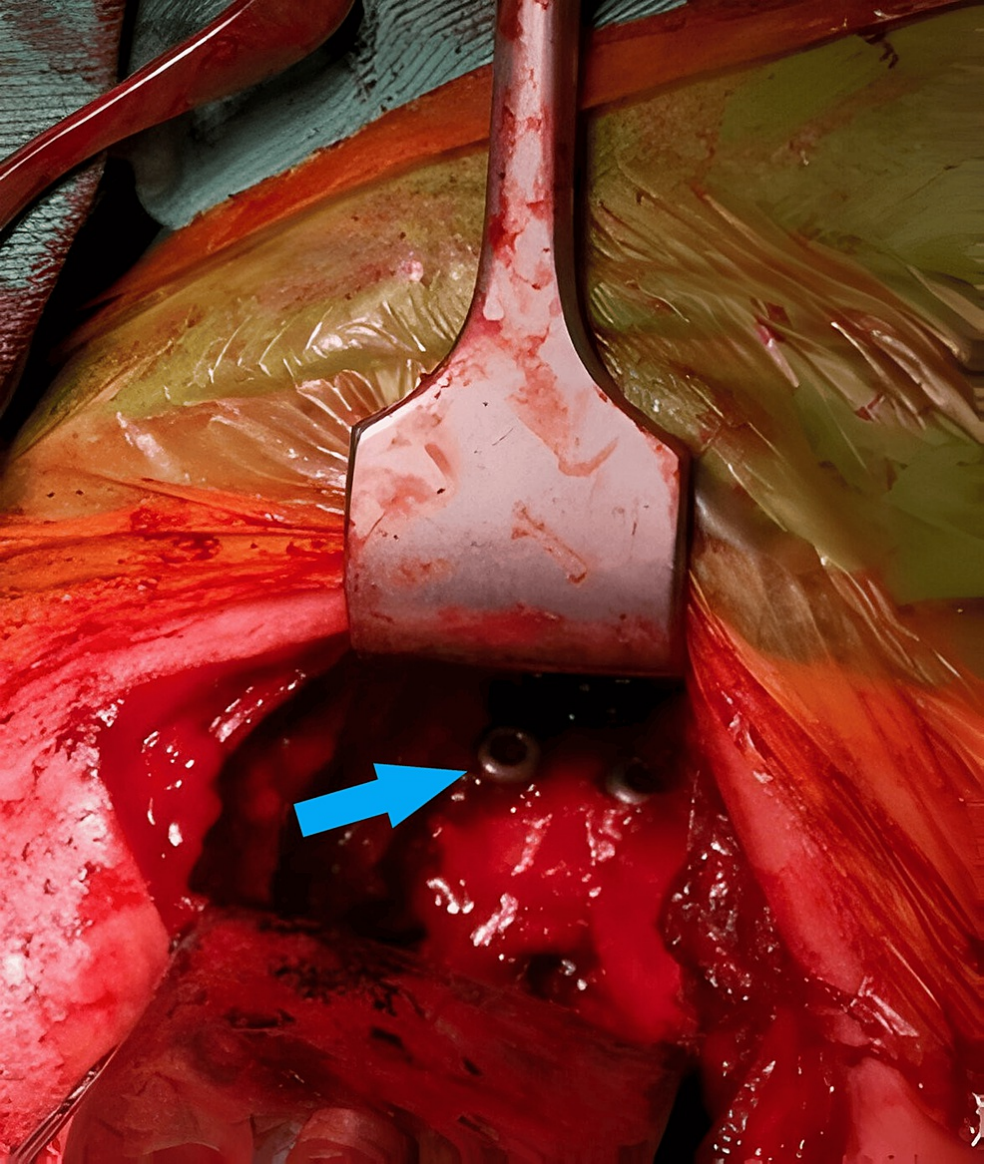
Intraoperative image showing screw fixation of the harvested coracoid process to the anterior glenoid (blue arrow).

The bilateral shoulder open Latarjet procedure involves transferring the coracoid process to the anterior glenoid rim, creating a bony block that prevents the anterior translation of the humeral head and provides stability to the shoulder joint (Figure 4). The surgery was performed sequentially, with the patient under general anesthesia, in a supine position propped up 30 degrees with a sandbag under the right shoulder. The Latarjet procedure was performed in a standard manner using the deltopectoral approach, with autograft coracoid bone blocks secured to the anterior glenoid rim using screws (Figure 5, 6). Additional procedures were carried out to address associated pathology, including labral repair and capsular plication.

**Figure 7:**
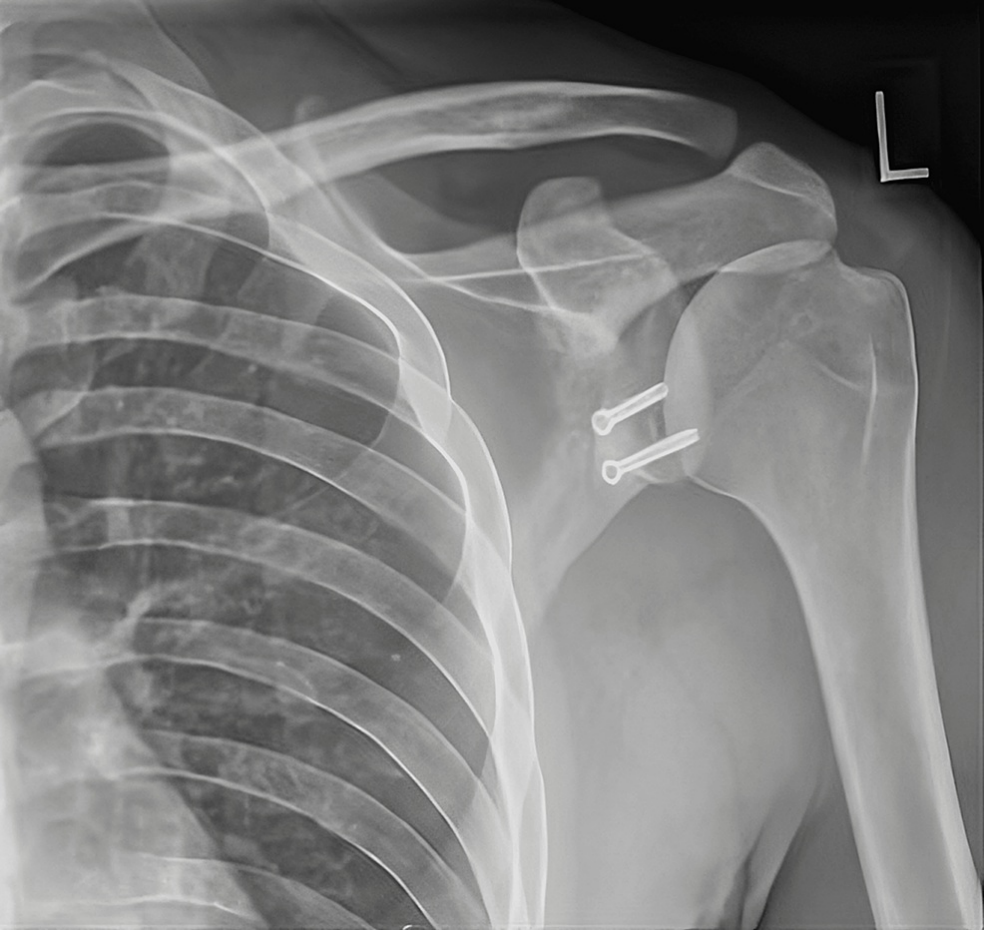
Radiograph (anterioposterior view) showing postoperative left shoulder Latarjet procedure.

**Figure 8:**
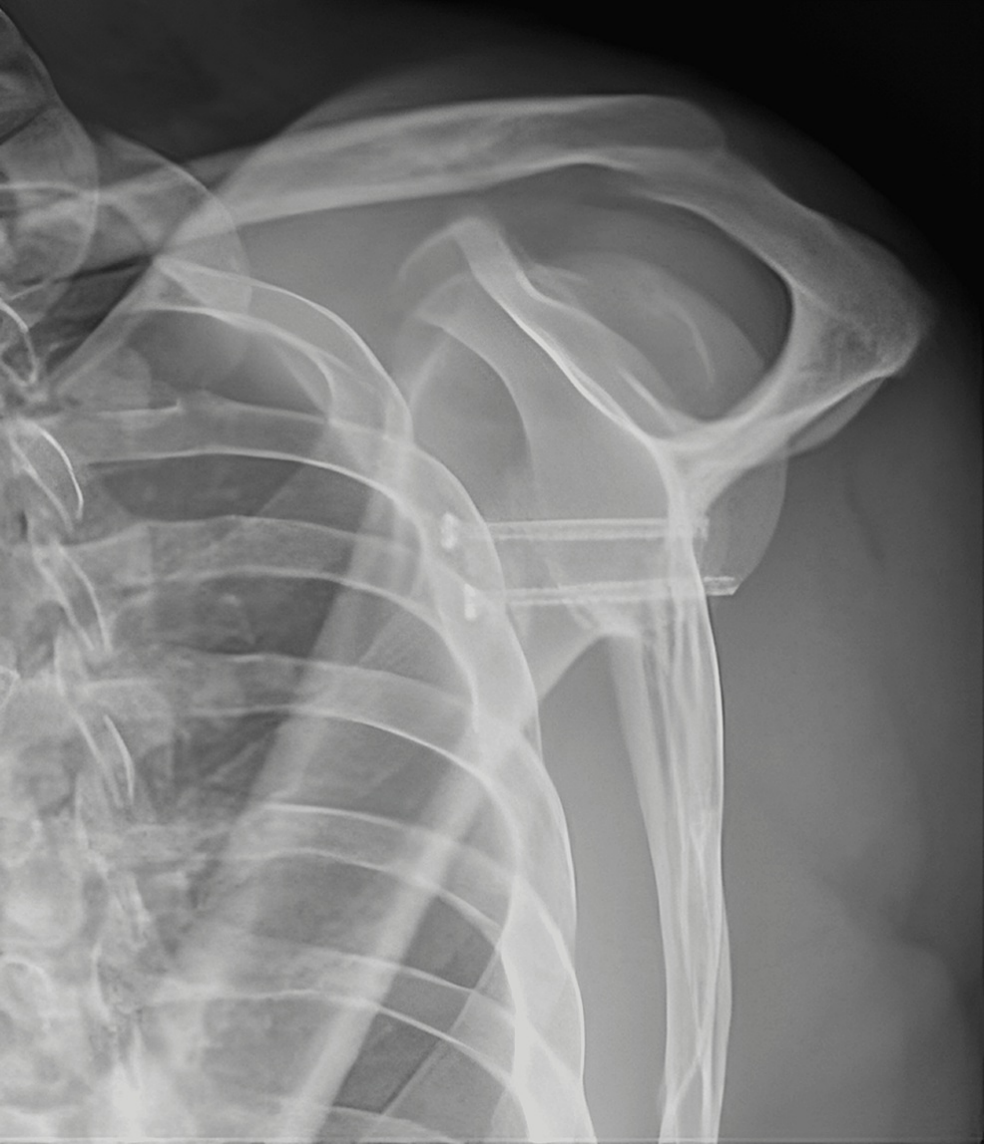
Postoperative radiograph (scapular Y view) showing left shoulder Latarjet procedure.

**Figure 9:**
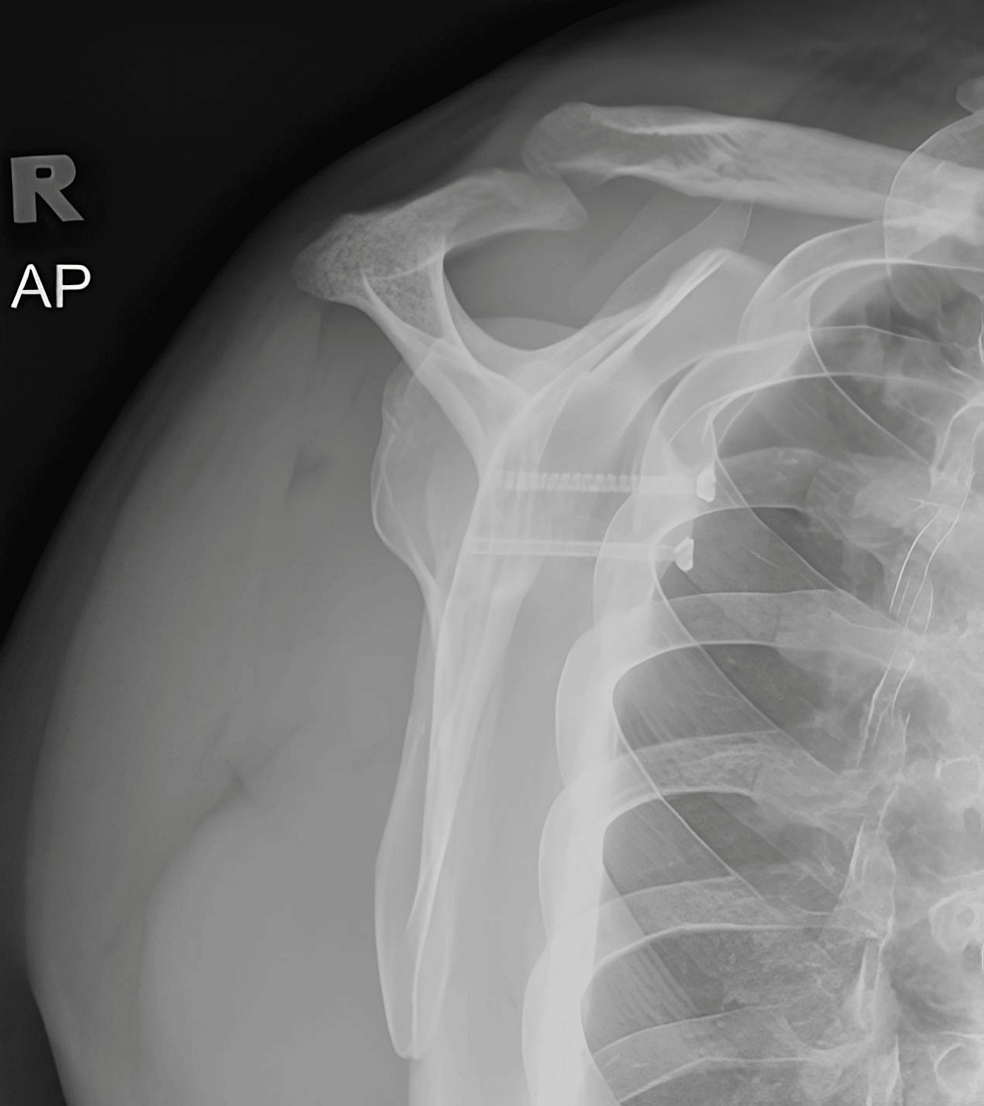
Postoperative radiograph (scapular Y view) showing right shoulder Latarjet procedure.

**Figure 10:**
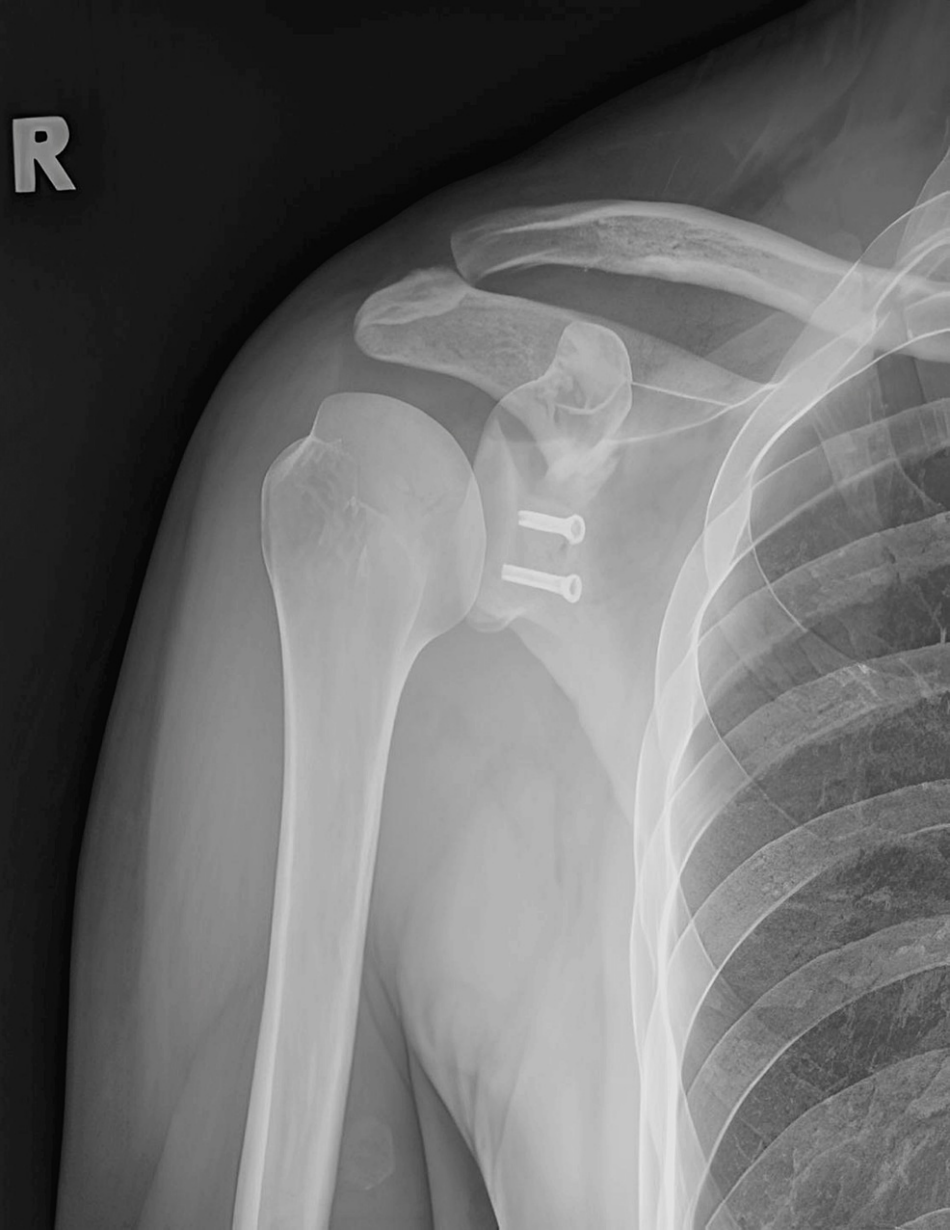
Radiograph (anterioposterior view) showing postoperative right shoulder Latarjet procedure.

Postoperatively, radiographs showed satisfactory results and the patient underwent a structured rehabilitation program (Figures 7-10). This program involved initial immobilization in shoulder slings followed by a progressive range of motion exercises, strengthening exercises, and proprioceptive training. The patient received regular follow-up evaluations to monitor progress, assess stability, and make necessary adjustments to the rehabilitation program.

**Figure 11:**
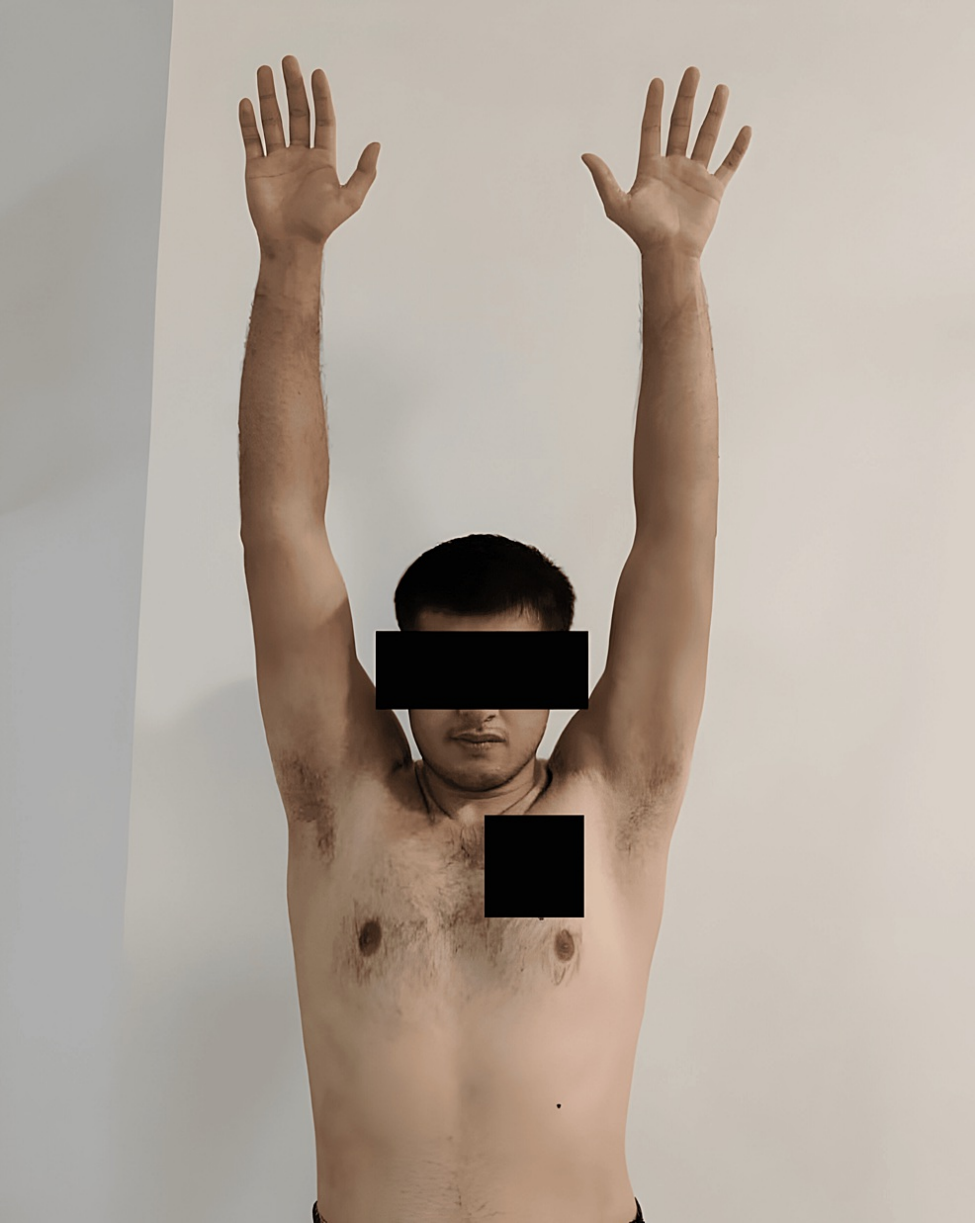
One-year postoperative image of patient being able to achieve full range of motion in shoulder forward flexion.

**Figure 12:**
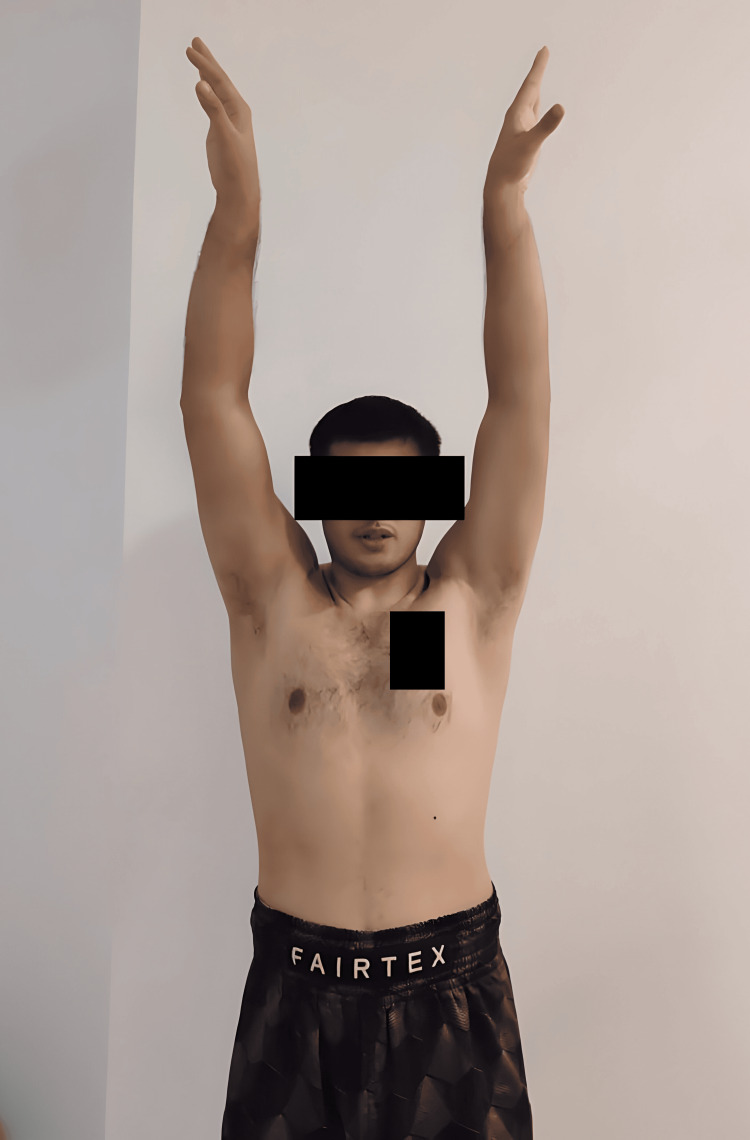
One-year postoperative image of patient being able to achieve full range of motion in shoulder abduction.

**Figure 13:**
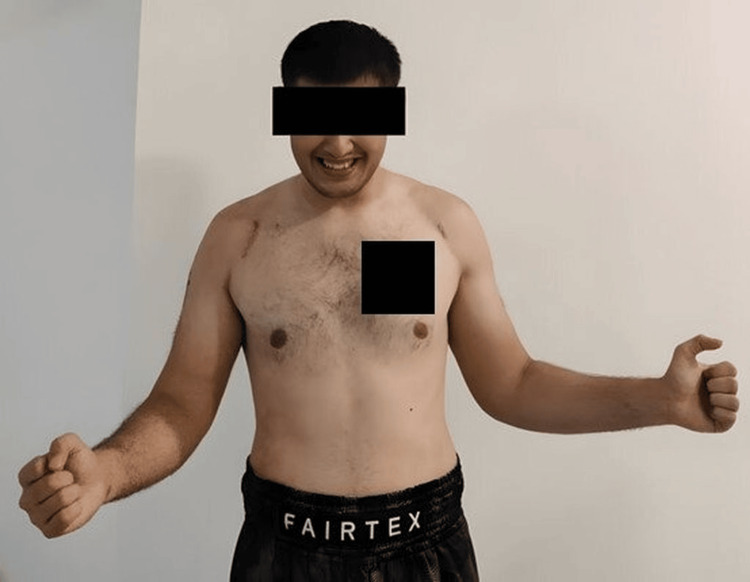
One-year postoperative image of patient being able to achieve about 60-70 degrees range of motion in bilateral shoulder external rotation.

At the one-year follow-up, the patient demonstrated substantial improvement in both shoulders. He reported no recurrence of dislocation or instability, enabling him to resume his role as a boxing instructor and engage in physical activities without any restrictions. The range of motion and strength in both shoulders exhibited remarkable enhancement compared to the preoperative condition. Specifically, the patient attained a full range of motion in shoulder forward flexion and abduction and nearly achieved a full range of motion in bilateral shoulder external rotation (Figures 11-13). Moreover, the patient expressed contentment with the surgical outcome and remains under our care for ongoing follow-up at the outpatient clinic.

## Discussion

Although bilateral shoulder dislocation is most often posterior, there are a few cases of bilateral anterior shoulder dislocation reported in the literature. They are the result of high-energy trauma, most often during high-speed sports accidents [1]. In young patients, like in the present case, the main complication of anterior shoulder dislocation is the instability of the shoulder; a prospective cohort study reported that 55.7% of young patients developed a recurrence of shoulder instability within two years [2]. Therefore, it's necessary to stabilize a young patient's shoulder with surgical treatment to prevent recurrent instability. Many options are possible but the most recommended are arthroscopic Bankart and the open Latarjet procedures [3].

The Latarjet procedure involves the transplant of the coracoid process to the scapular neck and has demonstrated excellent long-term clinical outcomes and return to sport rate. Recurrent instability is reported to be as low as 0-5.4% [4]. In our case, the open Latarjet technique was used with good clinical outcomes on bilateral shoulders. The Latarjet procedure is an established option in the treatment of recurrent anterior shoulder instability, and it’s particularly indicated in young, active patients with glenoid and/or humeral bone loss. The Latarjet procedure allows for a faster return to sports after surgery and most patients regain their preinjury level performance with good results [5,6].

The bone-blocking effect of the Latarjet procedure, achieved by fixing the coracoid graft flush with the joint line, compensates for anterior glenoid bone loss and increases the anterior-posterior diameter, resulting in glenoplasty. However, while this bony augmentation contributes to the stabilization provided by the Latarjet procedure, it is not the sole factor. Other factors that contribute to stability include the effect of the conjoined tendon acting as a sling on the inferior subscapularis and anteroinferior capsule when the arm is abducted and externally rotated, as well as the repair of the capsule to the coracoacromial ligament stump. The combined effect of bony, muscular, and capsular mechanisms, known as the "triple blocking effect" initially described by Patte and Debeyre, aims to minimize the occurrence of recurrent subluxation or dislocation [7].

Bilateral shoulder instability can be synchronous or asynchronous, depending on whether both shoulders are affected at the same time or at different times. In our case, the patient had asynchronous bilateral anterior instability due to traumatic events. The treatment of bilateral shoulder instability is challenging and requires a careful evaluation of the patient’s goals, expectations, and functional demands. The surgical options included simultaneous or staged procedures, arthroscopic or open techniques, and soft tissue or bone grafting procedures. The decision was made based on several factors, such as the type, direction, and severity of instability, the degree of glenoid bone loss, the presence of associated lesions, and the surgeon’s experience and preference.

A study conducted by Ernstbrunner and colleagues demonstrated that labral damage and greater glenoid bone loss had a substantial impact on increasing cartilage contact pressures in the shoulder, both on the glenoid and humeral sides [8]. While the Latarjet procedure could partially alleviate this effect, the positioning of the graft was found to be a crucial factor in determining the level of glenoid and humeral contact loading. In cases where there was a 25% loss of glenoid bone, performing the Latarjet procedure with a graft placed level with the glenoid and positioning the humerus at the midpoint of the glenoid led to a substantial rise in humeral cartilage contact pressure when compared to the preoperative condition [8].

Numerous research investigations have examined the comparison between arthroscopic Bankart repair with remplissage and the Latarjet procedure for individuals with off-track lesions and less than 25% glenoid bone loss [9]. These studies consistently reported similar outcomes in terms of patient-reported results, range of motion, pain levels, and rates of recurrence and return to sporting activities for both surgical methods. However, Yang and colleagues' findings indicated that collision athletes and those with more than 15% bone loss derived greater advantages from the Latarjet procedure in terms of patient-reported results, reduced instability recurrences, and lower revision rates, when compared to arthroscopic Bankart repair with remplissage [9].

Postoperative rehabilitation is a crucial component of the treatment plan following the bilateral shoulder open Latarjet procedure. A structured and progressive rehabilitation program is essential to optimize outcomes and facilitate the patient's return to function. Early range of motion exercises, followed by strengthening and stability exercises, are implemented to ensure proper graft healing, muscle activation, and joint coordination. Close collaboration between the orthopaedic team and physical therapist is vital to tailor the rehabilitation protocol to the patient's specific needs.

## Conclusions

This case report underscores the successful management of recurrent bilateral shoulder dislocation through a bilateral open Latarjet procedure. This surgical intervention significantly benefited the patient by facilitating an earlier return to sports activities. It proved to be an effective solution for addressing bilateral shoulder instability, resulting in favorable clinical outcomes, including stability restoration, increased range of motion, and enhanced functional capabilities. Additional research is necessary to assess the long-term effectiveness and compare outcomes between bilateral and unilateral approaches. Nevertheless, the bilateral open Latarjet procedure stands as a valuable treatment option for well-selected patients dealing with recurrent bilateral shoulder dislocation.
